# Comparative pharmacokinetics of fluralaner in dogs and cats following single topical or intravenous administration

**DOI:** 10.1186/s13071-016-1564-8

**Published:** 2016-05-31

**Authors:** Susanne Kilp, Diana Ramirez, Mark J Allan, Rainer KA Roepke

**Affiliations:** MSD Animal Health Innovation GmbH, Zur Propstei, 55270 Schwabenheim, Germany; ENVIGO CRS S.A., Centro Industrial Santiga, c/Argenters, 6, 08130 Santa Perpètua de Mogoda Barcelona, Spain

**Keywords:** Fluralaner, Dog, Cat, Pharmacokinetics, Bravecto™ Spot-on Solution

## Abstract

**Background:**

Bravecto™ Chewable Tablets for Dogs, containing fluralaner as active ingredient, is an innovative treatment for flea and tick infestations that provides safe, rapid and long acting efficacy after a single oral administration in dogs. Topically applied fluralaner provides similar safe, rapid and long acting efficacy, both in dogs and in cats. The pharmacokinetic profile of fluralaner was evaluated in dogs and in cats following either topical or intravenous administration.

**Methods:**

Twenty four dogs and 24 cats received three different topical doses, with the mid-dose based on the respective minimum recommended dose, and one intravenous dose. Plasma samples were collected for 112 days and fluralaner concentrations were quantified using a validated high performance liquid chromatography with tandem mass spectrometry (HPLC-MS/MS) method. Pharmacokinetic parameters were calculated using non-compartmental methods.

**Results:**

In dogs, fluralaner was readily absorbed from the topical administration site into the skin, subjacent tissues and blood. Fluralaner plasma concentrations showed an apparent plateau between ~ day 7 and 63, with individual t_max_ seen within this time period. After the plasma plateau, concentrations declined slowly and were quantifiable for more than 12 weeks. In cats, fluralaner was readily systemically absorbed from the topical administration site, reaching maximum concentrations (C_max_) in plasma between 3 and 21 days post administration, after which concentrations declined slowly, and were also quantifiable for more than 12 weeks. Systemic exposure, as shown by C_max_ and the area under the concentration versus time curve from time 0 to the last measurable concentration (AUC_(0→t)_) increased proportionally with dose in both species. Following intravenous administration fluralaner showed a relatively high apparent volume of distribution (V_z_), a low plasma clearance (Cl), a long terminal half-life (t_1/2_) and a long mean residence time (MRT); thereby demonstrating a long persistence of fluralaner in both species.

**Conclusions:**

The pharmacokinetic characteristics of fluralaner explain its prolonged activity against fleas and ticks on both dogs and cats after a single topical administration.

## Background

Bravecto™ Chewable Tablets for Dogs, containing fluralaner as active ingredient, is an innovative commercially available treatment for flea and tick infestations that provides safe, rapid and long acting efficacy after a single oral administration in dogs [1–4]. The active ingredient fluralaner belongs to the isoxazolines, a new class of compounds with potent antiparasitic activity. Fluralaner has activity against γ-aminobutyric acid- (GABA-) and glutamate-gated chloride channels with significant selectivity for insect neurons over mammalian neurons [5], which is predictive of the wide safety margin for this antiparasitic compound. Even at much higher than clinically relevant doses, fluralaner was well tolerated [2]. Fluralaner has a high potency against ticks and fleas by exposure via feeding, i.e. fleas and ticks that initiate feeding will be exposed to the active ingredient [6].

A newly developed spot-on formulation of fluralaner (Bravecto™ Spot-on Solution, MSD Animal Health) provides similar safe, rapid and long acting efficacy against fleas and ticks on dogs and cats. A single fluralaner dose administered topically at the minimum recommended dose of 25 mg/kg body weight (BW) on dogs and 40 mg/kg BW on cats provides at least 12 weeks of flea- and tick control. Only a short period of time is needed to effectively kill fleas (≤ 8 h) and ticks (≤ 12 h) on topically treated dogs [7] and cats. The fast onset together with the long duration of activity after a single topical administration offers a more convenient choice over monthly flea and tick control treatments that is expected to increase pet owner compliance. Increased compliance helps to reduce protection gaps that can occur with missed re-administration of monthly remedies.

Bravecto™ Spot-on Solution offers an opportunity for additional effective treatment. For example, the palatability of the chew was found to be 92.5 % [8], therefore a proportion of dogs may be easier to treat with a topical formulation; in addition, dogs that must be kept on allergen-restricted diets may be preferentially prescribed a topical formulation. Bravecto™ Spot-on Solution will be the only available isoxazoline-class active ingredient for cats providing 12 weeks of systemic protection against flea and tick infestations, thus satisfying an urgent need among owners and veterinarians for effective and reliable persistent ectoparasite control in cats.

The pharmacokinetic profiles of fluralaner in dogs and in cats following single topical administration describe the rate of absorption, distribution and elimination and provide insight crucial for understanding the onset, extent, and duration of efficacy.

Pharmacokinetic characteristics of topically applied fluralaner (Bravecto™ Spot-on Solution) were therefore investigated with a similar parallel group design in both dogs and cats.

## Methods

For both pharmacokinetic studies, animals were treated with the product, Bravecto™ Spot-on Solution, produced according to Good Manufacturing Practice [9]. Fluralaner for intravenous (i.v.) injection was formulated as 2.5 % solution in a PEG200 vehicle containing 10 % v/v water for injection.

All studies were compliant with the principles of Good Laboratory Practice [10]. The animal work was conducted in compliance with applicable national legislation and was reviewed and approved by the Animal Experimentation Ethics Committee at ENVIGO CRS S.A.

### In vivo phase

In the dog pharmacokinetic study 24 young healthy male and female Beagle (age 12–17 months; weight 7.4–12.1 kg) dogs were kept indoors in pens with sealed floors and individually housed for 5 weeks following topical fluralaner administration to avoid potential cross contamination between animals. After this 5-week period, dogs were penned in groups of 3 of the same treatment group and sex. In the cat pharmacokinetic study 24 young healthy male and female European short hair cats (age 9–11 months; weight 2.2–4.8 kg) were kept in individual cages to avoid potential cross contamination between animals.

During the time where animals were single housed, they had individual social contact with the caretaker, daily outside their pens/cages. Room environment was monitored continuously in both studies, with a temperature of 15–21 °C, relative humidity of 40–70 %, 10–20 air changes per hour and a 12-h fluorescent light/12-h dark cycle. Dogs and cats were fed once daily in the morning with a standard diet and had *ad libitum* access to water.

Dogs and cats were randomized to four treatment groups in each study (3 animals per sex per group), within sex, and blocked by body weight to ensure a balanced distribution.

One group of six animals was treated i.v. with a constant rate infusion over 5 min using an automatic injection system (KDS Model 200, KD Scientific Inc., Holliston, USA) at a fluralaner dose of 12.5 mg/kg BW. The perfusion rate per hour (mL/hr) was approximately 12 times higher than the respective dose volume to ensure complete administration within 5 min (dog i.v. data was previously published in Kilp et al. [4]).

All topical doses were calculated using individual body weights and the nominal content of fluralaner. In the dog study fluralaner (Bravecto™ Spot-on Solution) was administered topically to three groups of dogs at doses of 12.5, 25 or 50 mg/kg BW and was administered in one or several spots dorsally along the dogs’ back. The first spot was administered between the shoulder blades and the following spots, if necessary due to the treatment volume required, were administered approximately 5–10 cm caudal of the previously administered spot. In the cat study fluralaner (Bravecto™ Spot-on Solution) was administered topically at doses of 20, 40 or 80 mg/kg BW and was administered in one or several spots dorsally along the cat’s back. The first spot was administered at the base of the skull and the following spots, if necessary, were administered approximately 5–10 cm caudal of the previously administered spot.

Blood samples were collected from the jugular vein into sodium-citrate tubes before and at 1, 3, 5, 7, 10, 14, 21, 28, 35, 42, 49, 56, 63, 70, 77, 84, 91, 98, 105 and 112 days after topical administration and 15 min, 2, 4 and 8 h, and 1, 2, 3, 4, 7, 14, 21, 28, 35, 49, 63, 77, 91 and 112 days after i.v. dosing. Plasma was harvested by centrifugation and subsequently stored frozen (-75 °C ± 15 °C) in sterile plastic vials until analysis. The animals were closely observed for 1 h after dosing and once daily thereafter.

### HPLC MS/MS Analysis

Plasma concentrations of fluralaner were measured using a HPLC-MS/MS method, validated according to current guidelines [11–13]. Plasma samples were extracted by protein precipitation with acetonitrile and diluted with 0.1 % formic acid. The resultant solution was analysed quantitatively using automated solid phase extraction coupled to HPLC-MS/MS (online SPE-HPLC-MS/MS). The linear range of the method for determination of fluralaner was 10.0 to 2500 ng/ml, with a validated lower limit of quantification (LLOQ) of 10.0 ng/ml.

### Pharmacokinetic analysis

Pharmacokinetic parameters for fluralaner were calculated using non-compartmental methods with the validated software WinNonlin® Professional Version 5.3 (Pharsight Corporation, California, USA). C_max_ and time to peak concentration (t_max_) were observed values. The t_1/2_ was calculated by linear regression using the slope of the terminal segment of the semilogarithmic plasma concentration versus time curve. The AUC_(0→t)_ was calculated using the linear trapezoidal rule. The AUC from time 0 extrapolated to infinity (AUC_(0→∞)_) was determined as AUC_(0→ t)_ + Ct/λz, where Ct is the plasma concentration at time t and λ_z_ is the first order rate constant associated with the terminal (log-linear) portion of the curve. V_z_ after i.v. administration, based on the terminal phase was calculated as Dose/λ_z_ × AUC. Total body clearance (Cl) after i.v. administration was calculated using the ratio of Dose/AUC. Bioavailability (F %) via the topical route was calculated using mean AUC_(0→t)_ as (AUC_(0→t)_ topical/AUC_(0→t)_ iv) × (Dose iv/Dose topical) × 100. MRT extrapolated to infinity was calculated as the ratio of AUMC/AUC; where AUMC is the area under the first moment curve.

Dose proportionality was tested for exposure parameters C_max_ and AUC_(0→t)_. For this purpose dose-normalized (nominal dose) values were analysed using a one-way analysis of variance (ANOVA) where the exposure parameters are the dependent variable and the nominal dose is the independent variable. The test model null hypothesis was equal dose groups (level of significance α = 5 %); i.e. dose proportional increase in exposure parameters C_max_ and AUC_(0→t)_. If the null hypothesis cannot be rejected, there is no evidence against fluralaner dose proportionality.

All data are expressed as arithmetic means, unless otherwise stated. Individual values with a difference between AUC_(0→∞) _and AUC_(0→t)_ greater than 20 % of AUC_(0→∞)_ and/or an adjusted r^2^ (square of correlation coefficient) of the terminal phase regression line <0.85 were considered unreliable and therefore not taken into account for mean calculations. For graphical presentation of mean plasma concentrations of fluralaner, values below LLOQ (10 ng/ml) were set to ½ LLOQ (5 ng/ml), except when all values at a time point were below LLOQ.

## Results and Discussion

### Pharmacokinetic profile of fluralaner in dogs and in cats

In the pharmacokinetic study in dogs, each of four groups of six dogs was administered fluralaner topically at one of the three target doses (12.5, 25, or 50 mg/kg) or i.v. at a target dose of 12.5 mg/kg. In the pharmacokinetic study in cats, each of four groups of six cats was administered fluralaner topically at one of three target doses (20, 40, or 80 mg/kg) or i.v. at a target dose of 5 mg/kg. In both studies there were no clinical findings or adverse events following administration that could be related to fluralaner.

After topical administration on dogs, fluralaner penetrates rapidly into the skin and subjacent tissues in sufficient quantity to ensure fast onset of effective killing of fleas (≤ 8 h) and ticks (≤ 12 h) exposed to fluralaner by feeding [6, 7]. Repeated intensive shampooing beginning 3 days after topical administration did not impair flea and tick efficacy over 12 weeks [14]. As fluralaner has a low contact exposure activity compared to a high activity following parasite feeding exposure [6], this is consistent with rapid penetration into the skin and distribution to related tissue fluids and blood where fluralaner can exert its activity against ectoparasites, via feeding exposure.

Fluralaner plasma concentrations showed an apparent plateau between ~ day 7 and 63, with individual t_max_ seen within this time period.

**Fig. 1:**
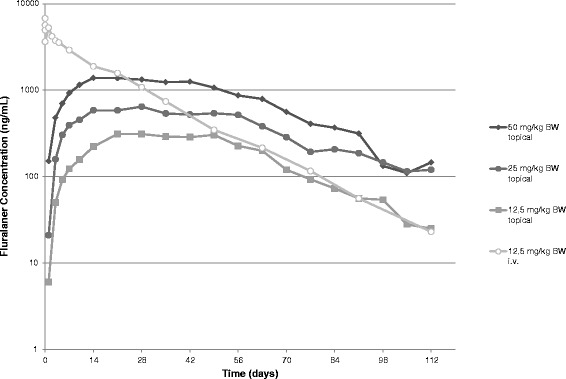
Mean plasma concentrations of fluralaner in dogs following single topical and i.v. administration

After the plasma plateau, fluralaner concentrations declined with quantifiable plasma concentrations detectable for more than 12 weeks post administration. Following i.v. administration the initial distribution of fluralaner was rapid, followed by a long elimination phase (Fig. 1). After both topical and i.v. administration some secondary peaks were present in the individual plasma concentration time profiles, which may indicate redistribution or recirculation e.g. enterohepatic re-circulation.

**Fig. 2:**
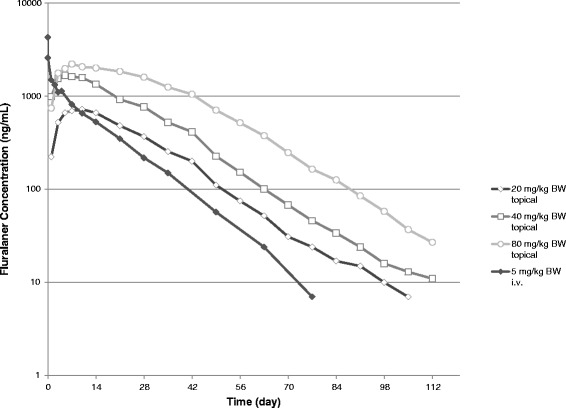
Mean plasma concentrations of fluralaner in cats following single topical and i.v. administration

In cats, following topical administration, fluralaner was readily absorbed, reaching individual maximum concentrations (t_max_) in plasma between 3 and 21 days. After the maximum concentration seen, fluralaner concentrations progressively declined with quantifiable plasma concentrations for more than 12 weeks post administration. Following i.v. administration fluralaner initial distribution was rapid, followed by a long elimination phase (Fig. 2).

Systemic bioavailability after topical administration was calculated to be ~25 % in both dogs and cats. Increases in the rate and extent of systemic exposure as shown by C_max_ and AUC_(0-t)_ appeared to be dose-proportional. No statistically significant differences between dose groups in dose-normalized exposure parameters AUC_(0→t)_ and C_max_ (ANOVA, *P* = 0.533 (dogs)/0.866 (cats) for AUC_(0→t)_ and *p*–0.388 (dogs)/0.254 (cats) for C_max_), indicated no evidence against the null hypothesis of fluralaner dose proportionality, thereby showing linear kinetics over the dose range of 12.5 to 50 mg/kg BW for dogs and of 20 to 80 mg/kg BW for cats.

Taking into account the total body water volume (approximately 0.6 L/kg in dogs and 0.5 L/kg in cats) [15], fluralaner shows a relatively high apparent distribution (V_z_ = 3.1 L/kg in dogs and 3.5 L/kg in cats) into tissues following i.v. infusion [4]. This is expected because the physicochemical properties of fluralaner with a molecular weight of 556.29, an unionized state at physiological pH (1–12), and a high logP_ow_ value of 5.35 [4, 5] favour the ability to cross cell membranes.

For fluralaner, the main route of elimination is likely hepatic because the high plasma protein binding [4] indicates minimal elimination via renal filtration. Plasma clearance can therefore be assumed to be equivalent to hepatic clearance. The clearance of fluralaner is low with only 0.14 L/kg/day in dogs [4] and 0.23 L/kg/day in cats. Considering a physiological hepatic blood flow of approximately 44.5 L/kg/day in the dog or 38.6 L/kg/day in the cat [15] and assuming hepatic clearance of fluralaner of 0.14 L/kg/day and 0.23 L/kg/day, respectively, the hepatic extraction ratio for fluralaner is estimated to be low (0.3 % in dogs [4] and 0.2 % in cats). The low clearance may be due to the high protein binding of fluralaner, which limits the unbound fraction of fluralaner in the vascular system that can be presented to clearing organs and/or due to a low intrinsic hepatic capacity to metabolize fluralaner [16–18].

As only unbound drugs in the vascular system are available to clearing organs for elimination, apparent volume of distribution (relatively high) and clearance (low) are determinants of the terminal half-life [16–18]. Accordingly, mean t_1/2_, but also the MRT of fluralaner were long. In both species, fluralaner could be quantified in plasma for more than 12 weeks post administration, demonstrating its long systemic persistence (Figs. 1 and 2).

The terminal plasma half-life was comparable following topical and i.v. administration, indicating that the terminal fluralaner plasma concentrations represented a true elimination phase. MRT after topical administration was considerably longer compared to i.v. administration due to the prolonged absorption phase.

The non-compartmental pharmacokinetic parameters calculated from the concentration–time data of fluralaner are shown for dogs (Table 1) and cats (Table 2).

**Table 1:** Mean (arithmetic) plasma pharmacokinetic parameters for fluralaner in dogs following single topical and i.v. administration

Parameters	Topical	Topical	Topical	Intravenous^b^
	12.5 mg/kg	25.0 mg/kg	50.0 mg/kg	12.5 mg/kg
	*n* = 6	*n* = 6	*n* = 6	*n* = 6
C_max_ (ng/mL)	358 ± 94	727 ± 191	1698 ± 318	7109 ± 908
t_max_^a^ (day)	42 (range 21–63)	25 (range 14–56)	25 (range 7–42)	n/a
AUC_(0→t)_ (day*ng/ml)	18933 ± 3599	41243 ± 7467	85852 ± 17283	87198 ± 11835
AUC_(0→∞)_ (day*ng/ml)	19577 ± 3982	43375^c^ ± 8752	93468^c^ ± 20424	87779 ± 12004
t_1/2_ (day)	17 ± 4	21^c^ ± 3	17^c^ ± 3	15 ± 2
t_last_ (day)	112 ± 0	112 ± 0	112 ± 0	109 ± 9
MRT (day)	47 ± 1	48^c^ ± 4	43^c^ ± 6	20 ± 3
Cl (L/kg/day)	n/a	n/a	n/a	0.14 ± 0.02
V_z_ (L/kg)	n/a	n/a	n/a	3.1 ± 0.5
F (%)	22 ± 4	24 ± 4	25 ± 5	n/a

^a^Median, other values are mean ± standard deviation

^b^Data previously published in Kilp et al. [4]; ^c^Mean consits of *n* = 4 (50.0 mg/kg)/ *n* = 5 (25.0 mg/kg) values; other values excluded as considered unreliable; see Methods

n/a - not applicable

**Table 2:** Mean (arithmetic) plasma pharmacokinetic parameters for fluralaner in cats following single topical and i.v. administration

Parameters	Topical	Topical	Topical	Intravenous
	20.0 mg/kg	40.0 mg/kg	80.0 mg/kg	5.0 mg/kg
	*n* = 6	*n* = 6	*n* = 6	*n* = 6
C_max_ (ng/ml)	757 ± 328	1850 ± 808	2399 ± 865	4302 ± 435
t_max_^a^ (day)	9 (range 5–10)	6 (range 3–14)	9 (range 7–21)	n/a
AUC_(0→t)_ (day*ng/ml)	22065 ± 7996	48161 ± 15294	89193 ± 21413	21863 ± 2160
AUC_(0→∞)_ (day*ng/ml)	22276 ± 7996	48400 ± 15177	89690 ± 21479	22141 ± 2105
t_1/2_ (day)	13 ± 2	12 ± 4	12 ± 1	11 ± 1
t_last_ (day)	93 ± 14	102 ± 12	110 ± 6	68 ± 7
MRT (day)	24 ± 3	23 ± 7	29 ± 5	15 ± 1
Cl (L/kg/day)	n/a	n/a	n/a	0.23 ± 0.02
V_z_ (L/kg)	n/a	n/a	n/a	3.5 ± 0.5
F (%)	25 ± 9	28 ± 9	25 ± 6	n/a

^a^Median, other values are mean ± standard deviation

n/a - not applicable

### Comparison of the pharmacokinetic profile in dogs and cats

The pharmacokinetic profiles of cats and dogs are both characterized by a long persistence of fluralaner. This is due to a high plasma protein binding, relatively high volume of distribution and a very low clearance in both species resulting in a long terminal half-life (11 days in cats and 15 days in dogs) and MRT (15 days in cats and 20 days in dogs) of fluralaner.

Maximum fluralaner concentrations in plasma were similar in dogs and cats at comparable topical dose levels (C_max_ 727 ng/ml in dogs at 25 mg/kg BW compared to 757 ng/ml in cats at 20 mg/kg BW).

Although C_max_ was similar at comparable topical dose levels in both species, the extent of systemic exposure to fluralaner in cats, as indicated by AUC, was approximately half that in dogs. The plasma concentration profile in the dog had an extended plateau between ~ day 7 and 63, whilst in cats T_max_ was seen sooner, followed by a longer and slightly steeper terminal elimination phase, indicating both faster absorption and faster elimination of fluralaner. This is also reflected in the shorter terminal half-life and MRT values in the cat after topical administration (12–13 and 23–29 days) compared to the dogs (17–21 and 43–48 days).

These results support the higher minimum recommended topical fluralaner dose in cats (40 mg/kg BW) compared with dogs (25 mg/kg BW) that delivers 12 weeks of flea- and tick control.

## Conclusion

The pharmacokinetic characteristics of fluralaner explain its prolonged activity against fleas and ticks on both dogs and cats after a single topical administration.

### Compliance statement

This study was conducted in Spain after obtaining the authorization of the relevant regulatory authorities.
